# Universal logic-in-memory cell enabling all basic Boolean algebra logic

**DOI:** 10.1038/s41598-022-24582-y

**Published:** 2022-11-22

**Authors:** Eunwoo Baek, Kyoungah Cho, Sangsig Kim

**Affiliations:** 1 Department of Semiconductor Systems Engineering, Korea University, 145 Anam-Ro, Seongbuk-Gu, Seoul, 02841 Republic of Korea; 2 Department of Electrical Engineering, Korea University, 145 Anam-Ro, Seongbuk-Gu, Seoul, 02841 Republic of Korea

**Keywords:** Electrical and electronic engineering, Nanoscale devices

## Abstract

Among the promising approaches for implementing high-performance computing, reconfigurable logic gates and logic-in-memory (LIM) approaches have been drawing increased research attention. These allow for improved functional scaling of a chip, owing to the improved functionality per unit area. Although numerous studies have been conducted independently for either reconfigurable logic or LIM units, attempts to construct a hybrid structure based on reconfigurable logic and LIM units remain relatively rare. In this study, we merge reconfigurable logic gates and LIM units to achieve a universal logic-in-memory (ULIM) cell for enabling all basic Boolean logic operations and data storage in a single cell. A ULIM cell consisting of silicon memory devices with reconfigurable n- and p-program modes can reconfigure logic operations within the complete set of Boolean logic operations. Moreover, the ULIM cell exhibits memory behaviors for storing output logic values without supply voltages for a certain period, resulting in zero static power consumption. Hence, this study provides a way to realize high-performance electronics by utilizing the silicon devices with a hybrid function of reconfigurable logic and LIM.

## Introduction

The demand for high-performance electronic hardware with high energy efficiency has been increasing, owing to the data explosion caused by the spread of artificial intelligence technologies^1^. However, conventional von Neumann-based computing architectures inevitably cause data bottlenecks due to the speed gap between the processor and memory units, and this data bottleneck intensifies as the amount of data increases. Aiming to overcome the limitations of conventional computing, numerous novel computing methods for replacing the von Neumann architecture have been researched in recent years^2–8^. Among them, reconfigurable logic gates and logic-in-memory (LIM) approaches have been of great interest as promising candidates for next-generation computing^7,9–15^.

Unlike complementary metal–oxide–semiconductor (CMOS) logic circuits, reconfigurable logic gates implement various logic operations in the same circuit structure, using elements capable of switching the channel type. This device-level reconfiguration enables the implementation of functionally improved logic gates with fewer resources^16–19^, thereby providing innovative solutions for performance improvements in traditional CMOS-based hardware facing limitations in dimensional scaling^20–22^. Whereas reconfigurable logic focuses on diversifying the functions in logic blocks, the scope of LIM research encompasses both logic and memory blocks and aims to merge logic calculations and data storage into one space^23,24^. This architectural convergence offers the possibility of high-performance hardware platforms suitable for large-capacity data processing, e.g., by increasing the integration density and eliminating the power consumption and latency caused by data movement between the processor and memory units.

Research on reconfigurable logic gates and LIM has mainly been conducted based on emerging memories such as resistive random-access memories (ReRAMs)^25,26^, magnetic RAMs (MRAMs)^27,28^, ferroelectric field-effect-transistors (FeFETs)^29–31^, or two-dimensional (2D) material transistors^10–12^. Reconfigurable logic gate and LIM concepts have been researched actively and independently from devices to architecture. Nevertheless, attempts to integrate these two concepts have generally been lacking^32^. Thus, there is a need for research on silicon-based technologies capable of merging reconfigurable logic gates and LIM units.

In this study, we merge reconfigurable logic gates and LIM units to construct a universal logic-in-memory (ULIM) cell for performing all basic logic operations and memorizing the output logic in a single cell. Triple-gate feedback random access memories (FBRAMs) are used to construct the ULIM cell, and each device has a p–i–n silicon nanowire structure with three gates for implementing the channel reconfiguration. A positive feedback mechanism, i.e., the operating principle of the triple-gate FBRAM, allows the device to act as a memory element based on the accumulation of charge carriers in the channel^33,34^. The ULIM cell can perform eight Boolean logic operations (including those of XNOR and XOR logic gates) in one cell by using the channel reconfiguration characteristics of the triple-gate FBRAMs. Moreover, the ULIM cell exhibits excellent memory characteristics; for example, it maintains an output logic value for a certain period under zero supply voltages. We demonstrate the logic reconfiguration and LIM operations of the proposed ULIM cell through a mixed-mode technology computer-aided simulation, demonstrating the potential for realizing high-performance electronics with this novel structure.

## Methods

All simulations for a single device and mixed-mode simulations were conducted based on the 2D structure of the triple-gate FBRAM using a commercial device simulator (Synopsys Sentaurus (O_2018.06))^35^. The physics model for the triple-gate FBRAM included the Fermi–Dirac statistics and Slotboom bandgap narrowing. The recombination mechanism followed Shockley–Read–Hall recombination, with a doping dependency and Auger recombination. To analyze the silicon region, we used high-field saturation mobility and inversion and accumulation layer mobility models to consider the doping and transverse-field dependencies for the 2D Coulomb impurity scattering. Additionally, we added surface Shockley–Read–Hall recombination to the interface between the silicon and Al_2_O_3_ in the triple-gate FBRAM.

## Results and Discussions

### Design and electrical characteristics of triple-gate feedback random access memory (FBRAM)

**Figure 1:**
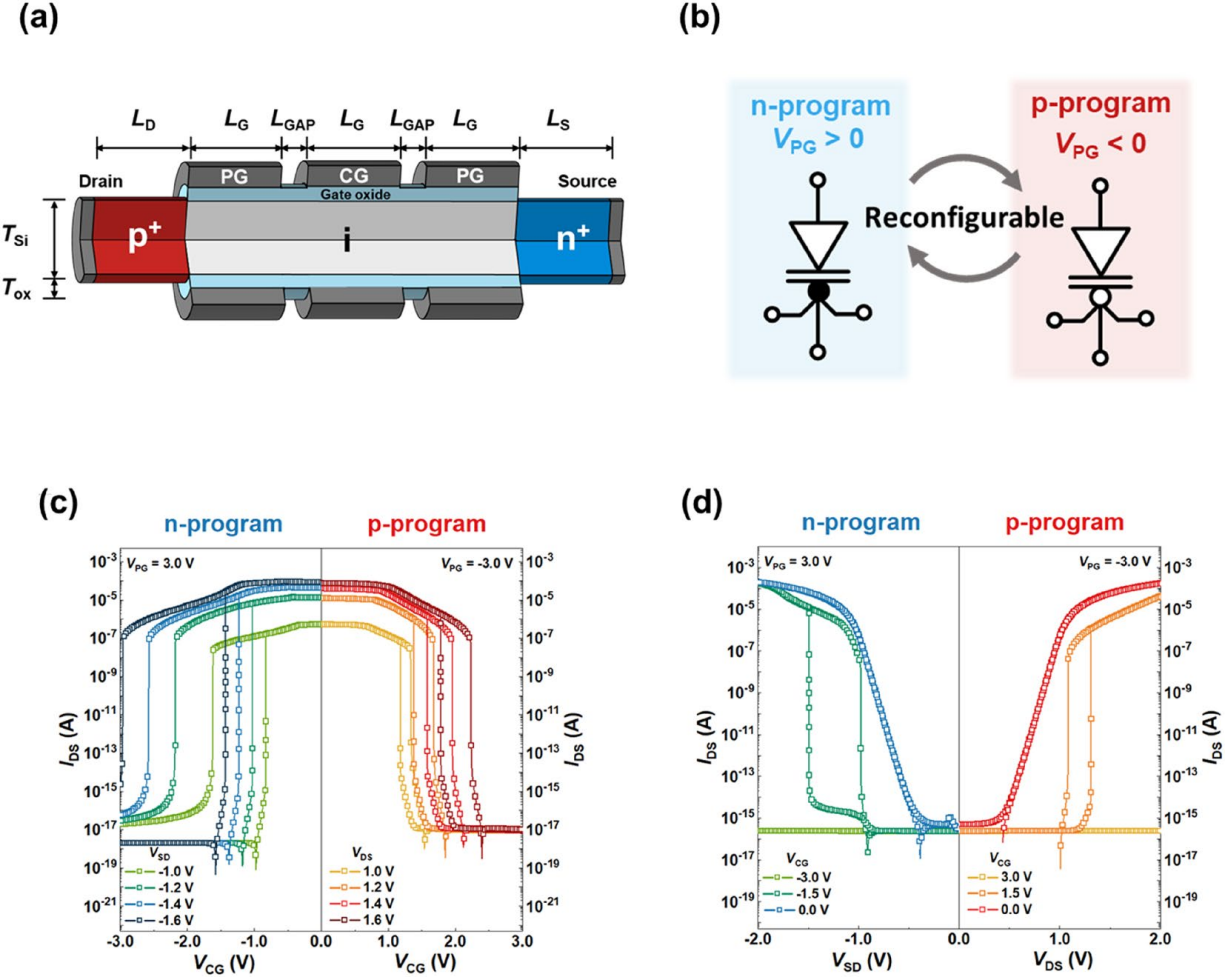
Design and electrical characteristics of triple-gate feedback random access memory (FBRAM) (**a**) Schematic of triple-gate FBRAM. (**b**) Circuit symbols of triple-gate FBRAM when operating in n- and p-program modes. (**c**) Transfer characteristics of triple-gate FBRAM in n- and p-program modes under several supply voltages. (**d**) Output characteristics of triple-gate FBRAM in n- and p-program modes under various *V*_CG_.

Figure 1A shows a schematic of the triple-gate FBRAM, which acts as a basic element of the ULIM cell. The triple-gate FBRAM has a p–i–n silicon nanowire structure, in which one control gate (CG) electrode is located between two program gate (PG) electrodes and can switch between n-/p-program modes depending on the polarity of the voltages applied to the PGs. The CG performs as a switching gate for turning the device on or off, and the logic input voltage (*V*_IN_) is input to the CG when extended to the circuit level. The gate length (*L*_G_) is 50 nm for all three gates, and the separation between the gates (*L*_GAP_) is 10 nm. The drain and source lengths (*L*_D_ and *L*_S_) are 50 nm, and the channel and oxide thicknesses (*T*_Si_ and *T*_OX_) are 10 nm and 2 nm, respectively. The p^+^ drain and n^+^ source regions are heavily doped with 1 × 10^20^ cm^-3^ and the intrinsic channel region is lightly p-type doped with 1 × 10^15^ cm^-3^. The work function of all gate electrodes is set to 4.8 eV. The device and circuits are designed and simulated based on a 2D structure, using a commercial device simulator (Synopsys Sentaurus)^35^. The triple-gate FBRAM operates in the n-program (p-program) mode when a positive (negative) programming voltage (*V*_PG_) is applied to the PG. To differentiate between the devices in accordance with the program mode more clearly in the circuit diagrams, circuit symbols have been devised for the triple-gate FBRAMs operating in the n- and p-program modes, as shown in Fig. 1b. As the two PGs are electrically coupled, the bias line for the PGs is expressed as one line in the circuit symbols. The symbol for an unprogrammed mode with a programming voltage (*V*_PG_) of 0.0 V is illustrated in Supplementary Fig. 1(a).

Figure 1c shows the transfer characteristics of the triple-gate FBRAM as operating in n- and p-program modes when various supply voltages are applied. *V*_PG_ values of 3.0 V and − 3.0 V are applied to operate the device in the n- and p-program modes, respectively. In both program modes, the triple-gate FBRAM exhibits bistable and steep switching characteristics, owing to the operating principle of the positive feedback loop^34,36^. When sweeping the control gate voltage (*V*_CG_) in the n-programmed (p-programmed) device from − 3.0 V (3.0 V) to 0.0 V, a rapid increase in the drain current (*I*_DS_) occurs at a specific *V*_CG_; this is defined as a latch-up voltage, due to the generation of a positive feedback loop. At an absolute drain-to-source voltage (*V*_DS_) value of 1.0 V, the device has extremely low subthreshold swings of 2.66×10^–3^ mV/dec for the n-program mode and 4.53×10^–2^ mV/dec for the p-program mode at the latch-up voltages. The device switches from the off state to the on state after the latch-up phenomenon and exhibits high on/off current ratios of over 10^11^ for the n-program mode and over 10^10^ for the p-program mode at |*V*_DS_|= 1.0 V. When sweeping *V*_CG_ from 0.0 V to the initial voltage values, *I*_DS_ decreases rapidly in both program modes at a specific *V*_CG_; this *V*_CG_ is referred to as a latch-down voltage. The latch-down phenomenon results from the elimination of the positive feedback loop. Here, the latch-up voltages are different from the latch-down voltages, and this voltage difference is defined as the memory window. When |*V*_DS_| increases, the latch-up/down voltages move in the negative (positive) voltage direction in the n-program (p-program) mode. Correspondingly, the memory window width increases, because the change in the latch-down voltage is substantially larger than that in the latch-up voltage. Moreover, the on current increases while the off current before the latch-up does not change, leading to an increase in the on/off current ratio. The n-programmed device exhibits a slight increase of the off current after the latch-down because of the remained carriers inside the device; however, the difference is less than ~ 10 times which is negligible in further applications or possible to be improved by optimizing the device structure. The width difference in the memory window in the n- and p-program modes originates from the mobility differences between electrons and holes. Figure 1d shows the output characteristics of the triple-gate FBRAM in the n- and p-program modes. When |*V*_CG_|= 3.0 V, the devices in both the n- and p-program modes remain in the off state while |*V*_DS_| increases from 0.0 V to 2.0 V. For |*V*_CG_|= 1.5 V, the devices show the bistable and steep switching characteristics, owing to the generation and elimination of the positive feedback loop. In contrast, the output characteristics exhibit the behaviors similar to those of a conventional p–i–n diode when |*V*_CG_|= 0.0 V, because the value of *V*_CG_ is not sufficient to build the potential barriers and wells for triggering the positive feedback loop^36^.

### Operating principle of the triple-gate FBRAM

**Figure 2:**
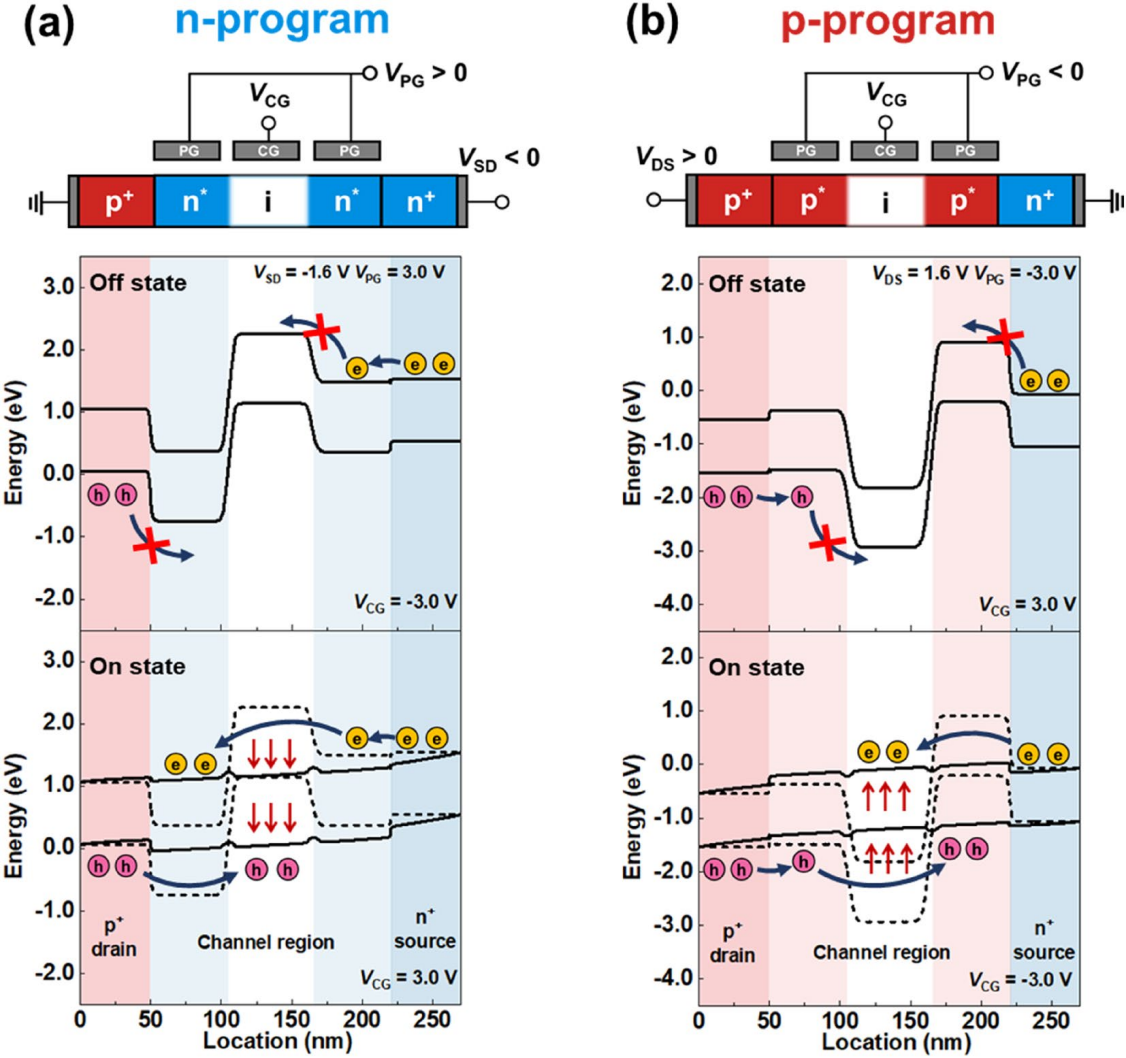
Operating principle of triple-gate FBRAM Energy band diagrams of triple-gate FBRAMs in on and off states of (**a**) n- and (**b**) p-program modes. According to polarity of *V*_PG_, potential barriers and wells are created in energy band diagrams for operating in the corresponding program mode. The potential barriers and wells play key roles in triggering the positive feedback loop.

Referring to Fig. 2, we can provide detailed explanations of the device characteristics based on energy band diagrams of the triple-gate FBRAM in the n- and p-program modes. As shown in Fig. 2a, when *V*_CG_ = − 3.0 V in the n-program mode with *V*_PG_ = 3.0 V and the drain-to-source voltage (*V*_DS_) =  − 1.6 V, potential barriers and wells are built in the energy bands in the form of p^+^–n^*^–i–n^*^–n^+^. Thus, the device is in the off state. n^*^ indicates that the corresponding channel region is electrostatically doped as an n-channel, owing to the positive *V*_PG_. Despite having the same *V*_PG_, each n^*^ region has different energy levels, because the electrons injected from the source region further increase the energy level in the n^*^ region adjacent to the source region. Notably, the energy levels of the corresponding n^*^ region cannot be higher than those of the source region, owing to the channel-pinning phenomenon^37^. While increasing the *V*_CG_ from − 3.0 V to 3.0 V, electrons in the source region flow into the channel region, as the potential barrier height for the electrons becomes lower. The accumulation of electrons inside the channel lowers the potential barrier height for the holes and allows the holes to flow from the drain region into the channel. The accumulation of holes in the channel lowers the potential barrier height for the electrons, and further activates the injection of electrons into the channel. This continuous reduction in the potential barrier height owing to the accumulation of charge carriers results in a positive feedback loop. Owing to this positive feedback loop, the device switches to the on state with a latch-up phenomenon. In Fig. 2b, when *V*_CG_ = 3.0 V in the p-program mode with *V*_PG_ = − 3.0 V and *V*_DS_ = 1.6 V, the potential barriers and wells are built in the energy bands in the form of a p^+^–p^*^–i–p^*^–n^+^ structure; thus, the device is in the off state. p^*^ indicates that the corresponding channel region is electrostatically doped as a p-channel, owing to the negative *V*_PG_. With a mechanism identical to that of the n-program mode, the energy levels of each p^*^ region show differences under the same *V*_PG_. In other words, the holes injected from the drain region lower the energy levels in the p^*^ region beside the drain, resulting in differences in the energy levels of each p^*^ region. The channel-pinning phenomenon is equally applied to the p-program mode. While decreasing the *V*_CG_ from 3.0 V to − 3.0 V, the continuous interactions between the charge carrier and potential barrier height induce the positive feedback loop; consequently, the device switches to the on state with the latch-up phenomenon. To generate the latch-down phenomenon for switching the device to the off state, the elimination of the positive feedback loop is required. This can be accomplished by applying a voltage sufficient to remove the charge carriers stored inside the channel. Because the device maintains the on state before eliminating the positive feedback loop, it has a memory window in the I–V curve. Therefore, the storage of the charge carriers inside the channel based on the positive feedback loop plays a key role in the functioning of the triple-gate FBRAM as a memory device.

### Universal logic-in-memory (ULIM) cell demonstrating XNOR/XOR logic gate operation

**Figure 3:**
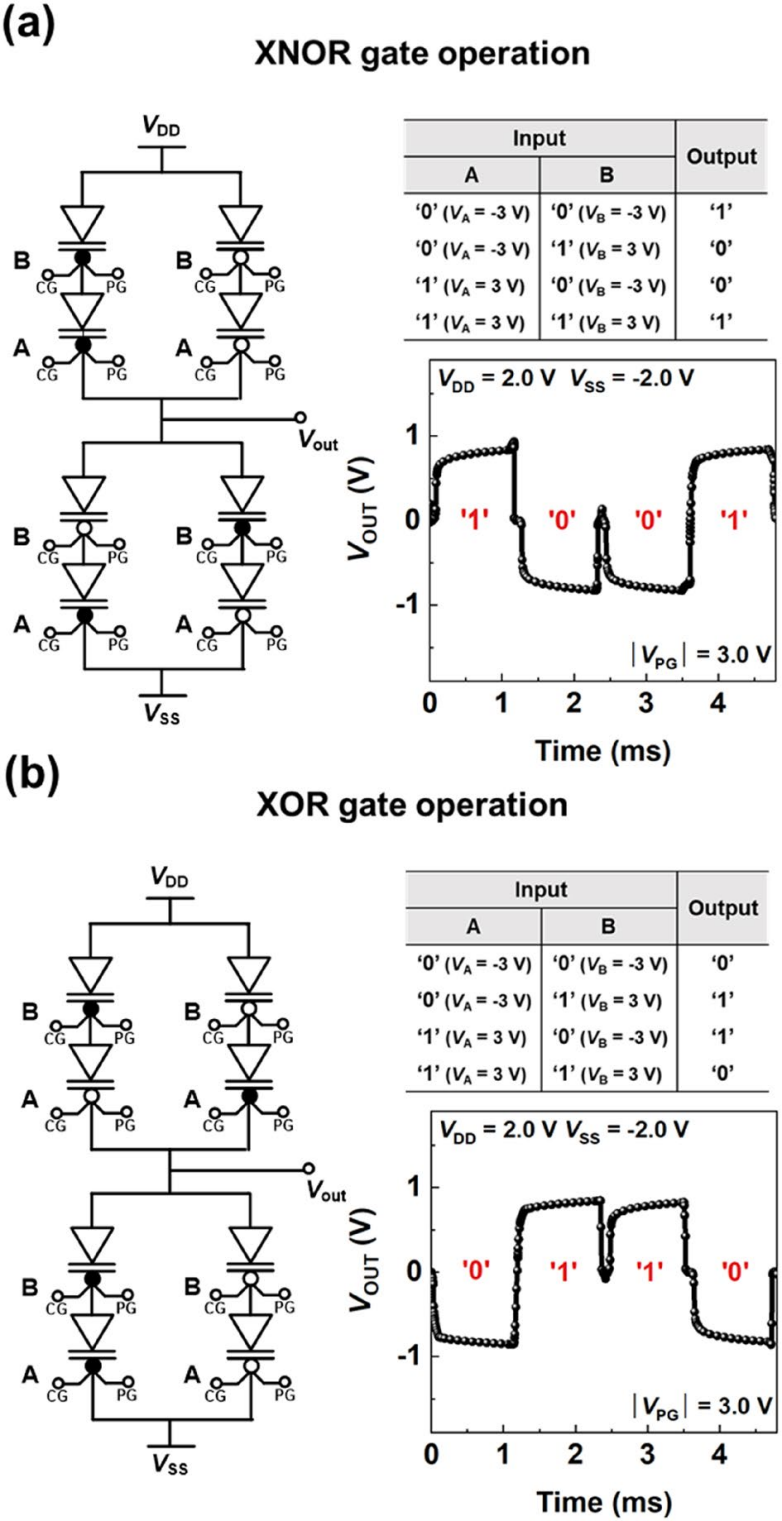
Universal logic-in-memory (ULIM) cell demonstrating XNOR/XOR logic gate operations. Circuit diagrams, truth table, and timing diagrams for (**a**) XNOR and (**b**) XOR logic gate operations.

The triple-gate FBRAM acts as a multifunctional device with memory behaviors and reconfigurable characteristics. Therefore, these multiple functions can be utilized more diversely when extended to the circuit level beyond a single element. In the ULIM cell, two serial connections consisting of two triple-gate FBRAMs are symmetrically connected based on the output node, as shown in Fig. 3. We considered a parasitic capacitance of 1fF when simulating the circuit behaviors.

The circuit diagram before programming each triple-gate FBRAM is shown in Supplementary Fig. 1(b). The value of *V*_PG_ is 3.0 V for the n-programmed device, and − 3.0 V for the p-programmed device, whereas *V*_DD_ and *V*_SS_ are maintained at 2.0 V and − 2.0 V, respectively. The voltages for the input logic values of ‘1’ or ‘0’ are 3.0 or − 3.0 V, respectively, and apply to the input nodes of A and B as input voltages A and B (*V*_A_ and *V*_B_). By altering the combinations of *V*_PG_ and *V*_IN_, the ULIM cell can perform all basic Boolean logic operations (such as NOT, YES, AND, OR, NAND, NOR, XOR, and XNOR) in a single cell. Herein, the XOR and XNOR logic operations are described as representative examples. As shown in Fig. 3a,b, the XNOR/XOR logic gate operations are implemented by programming transistor networks located at the top and bottom of the output node and applying input voltages. The other logic gate operations are performed equally under the XNOR/XOR logic gate conditions, as shown in Supplementary Fig. 2. As the XNOR and XOR logic gates have opposite output logic values, the top and bottom networks have opposite program configurations. This applies equally to NOT/YES, NAND/AND, and NOR/OR relationships.

When the input logic values are applied to two corresponding input nodes, a current path between *V*_OUT_ and *V*_DD_ or *V*_SS_ is created, according to the program state and arrangement of the device. In the case of XNOR logic gates, when the input logic ‘00’ is applied to nodes A and B, the p-programmed devices arranged in series in the top network create a path between *V*_DD_ and *V*_OUT._ However, in the bottom network, the current path between *V*_SS_ and *V*_OUT_ is disconnected, because devices with different program modes are serially connected. Therefore, the output logic value of the XNOR logic gate for the input logic ‘00’ is ‘1’. In the same manner, applying the input logic values of ‘01’, ‘10’, and ‘11’ to nodes A and B of the XNOR logic gate results in output logic values of ‘0’, ‘0’, and ‘1’, respectively. For the XOR logic gate, applying the input logic of ‘00’ to nodes A and B creates a current path between *V*_OUT_ and *V*_SS,_ as the n-programmed devices are arranged in series in the bottom network; the current path between *V*_DD_ and *V*_OUT_ in the top network is blocked by the serial connections of the different programmed devices. Therefore, the output logic of the XOR logic gate for the input logic value of ‘00’ is ‘0’, and the input logic values of ‘01’, ‘10’, and ‘11’ to nodes A and B of the XOR logic gate result in output logic values of ‘1’, ‘1’, and ‘0’, respectively. Note that the *V*_OUT_s of around ± 1 V are exhibited as the result of the voltage division between the *V*_DD_ and *V*_SS_ because the FBRAM functions as a memory element with capacitance components embedded within the channel. However, the voltages of the logic input and output were mismatched in the ULIM cell, which lowers the ability of the ULIM circuit to drive the next circuit. A voltage amplifier or other devices are needed to eliminate the mismatch. The logic cascading issue remains in our future research. In contrast to CMOS logic circuits that require over 20 transistors each to implement XNOR and XOR logic gate operations, our ULIM cells can implement logic operations with fewer devices and can freely reconfigure logic operations by simply changing the electrical signals. This logic reconfiguration capability presents the potential of the ULIM cell that can be utilized as a function unit in hardware-level programmable arrays with excellent energy efficiency and functionality per chip. For an ULIM cell array, the combinational logics such as a half-adder and a decoder can be implemented by programming individual cells.

In Fig. 3, the *V*_OUT_ becomes zero in the middle of the transition from one output logic into another. For the FBRAMs in the ULIM cell (at *V*_DD_ = 2.0 V and at *V*_SS_ = − 2.0 V), the generation of the positive feedback loop (at *V*_IN_ = 3.0 V) stimulates the charging (accumulation) of charge carriers in the channels and the elimination of the positive feedback loop (at *V*_IN_ = − 3.0 V) leads to the discharging (extinction) of the charge carriers from the channels^34,36^. The accumulation of charge carriers in the channels and the extinction of the charge carriers from the channels create the on and off states of the FBRAMs, respectively. For the FBRAMs, the discharging time is longer than the charging time when *V*_DD_ and *V*_SS_ are maintained at 2.0 V and − 2.0 V, and thereby the switching from the on state into the off state takes a longer time than that from the off state into the on state. Consequently, during the XOR and XNOR logic operations, the states of all the FBRAMs in the ULIM cell become the on state in the middle of the transition from one output logic to another. The FBRAMs in the on state have the internal channel resistance and thereby the programming transistor networks located at the top and bottom of the output node have the same resistance when the states of all the FBRAMs in the ULIM cell become the on state. Therefore, the *V*_OUT_ becomes zero in the middle of the transition from one output logic into another.

### Logic-in-memory (LIM) operation of the ULIM cell

**Figure 4:**
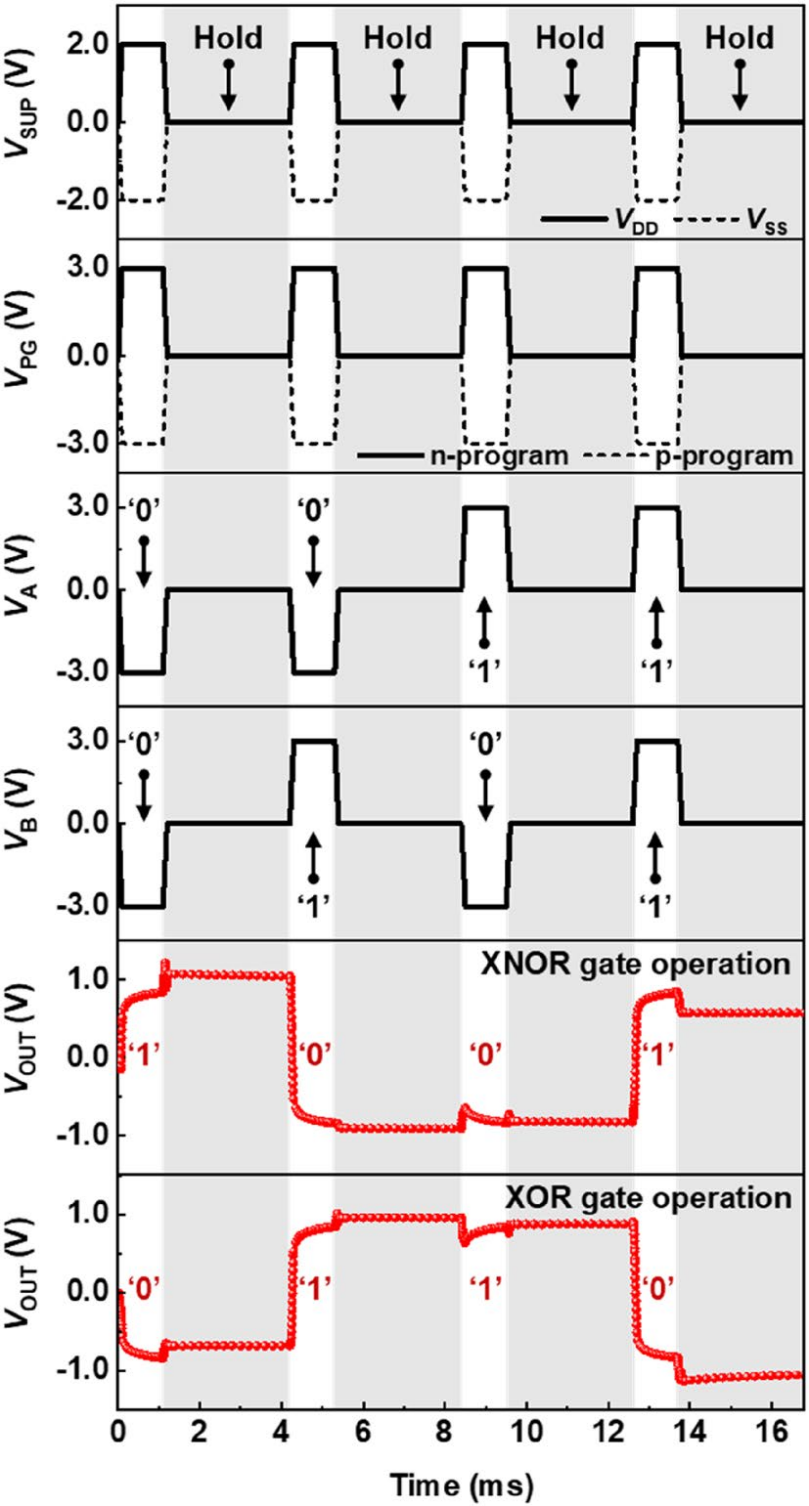
Logic-in-memory (LIM) operation of the ULIM cell. Timing diagrams when applying repeated pulses for XNOR and XOR logic gate operations.

Recently, the study of quasi-nonvolatile memories utilizing a device operated by positive feedback loops has been proposed with high speed and long retention time of 100 s under the zero supply voltages. Accordingly, it is crucial to check the memory characteristics as well as the logical operation of the ULIM cell. We can examine the LIM operations of the ULIM cell by repeating the logic and hold operations. The timing diagrams in Fig. 4 show the LIM operations of the ULIM cells for the XNOR and XOR logic gates. The logic calculations are performed by applying the supply voltages (*V*_SUP_), *V*_PG_, *V*_A_, and *V*_B_ for 1 ms; these supply voltages have the same voltage values as the aforementioned logic operations. Subsequently, the hold operations for verifying the memory behavior of the ULIM cell are retained for 3 ms by setting *V*_SUP_, *V*_PG_, *V*_A_, and *V*_B_ to zero. The LIM operations for other logic gates are shown in Supplementary Fig. 3. The cell programmed as an XNOR logic gate calculates the corresponding output logic values of ‘1’, ‘0’, ‘0’, and ‘1’ when the input logic values of ‘00’, ‘01’, ‘10’, and ‘11’ are applied, respectively. After the logic operation, the output voltages are maintained at a level comparable to that of the initial output logic value during the hold operation, thereby manifesting memory behavior. For the XOR logic gate operation, the cell provides the corresponding output logic values of ‘0’, ‘1’, ‘1’, and ‘0’ when the input logic values of ‘00’, ‘01’, ‘10’, and ‘11’ are applied, respectively. The ULIM cell retains the output voltages at levels comparable to those of the initial output logic values during the hold operation, without any external bias. This output logic retention characteristic of the ULIM cell is owing to the excellent charge storage capability of triple-gate FBRAM, i.e., the basic element of the cell. The charge carriers injected during the logic calculations remain within the channel region even the zero external bias conditions, allowing the triple-gate FBRAM to operate as a memory device for a certain period under zero supply voltages^36^. Accordingly, the *V*_OUT_ of the ULIM cell can be maintained during the hold operation, owing to the quasi-nonvolatile characteristics of the single device. Because of the logic retention capability from storing the logic states as the value of *V*_OUT_, the power consumption of the ULIM cell during the hold operation is zero. Additionally, the short circuit current of around 5×10^–13^ A flows through the circuit on average while transitioning the logic state. Therefore, the dynamic power consumption of ULIM cell is calculated as an average of around 2 pW to calculate each output logic for the input logic.

In Fig. 4, the *V*_OUT_ does not come back to zero, owing to the hold operation (at *V*_DD_ = 0.0 V and at *V*_SS_ = − 0.0 V) prior to the transition from one output logic to another (see Fig. 3). For the FBRAMs, the switching time from the on state in the hold operation into the off state is shorter than that from the on state (at *V*_DD_ = 2.0 V and at *V*_SS_ = − 2.0 V) into the off state. And the switching times from the on state in the hold operation into the off state is not longer than that from the off state in the hold operation into the on state. Thus, the case that the states of all the FBRAMs in the ULIM cell become the on state in the middle of the transition from one output logic in the hold operation to other output logic does not happen. Consequently, during the XOR and XNOR logic operations, the programming transistor networks located at the top and bottom of the output node have the different resistance, and the *V*_OUT_ does not come back to zero.

### Logic retention characteristics of the ULIM cell

**Figure 5:**
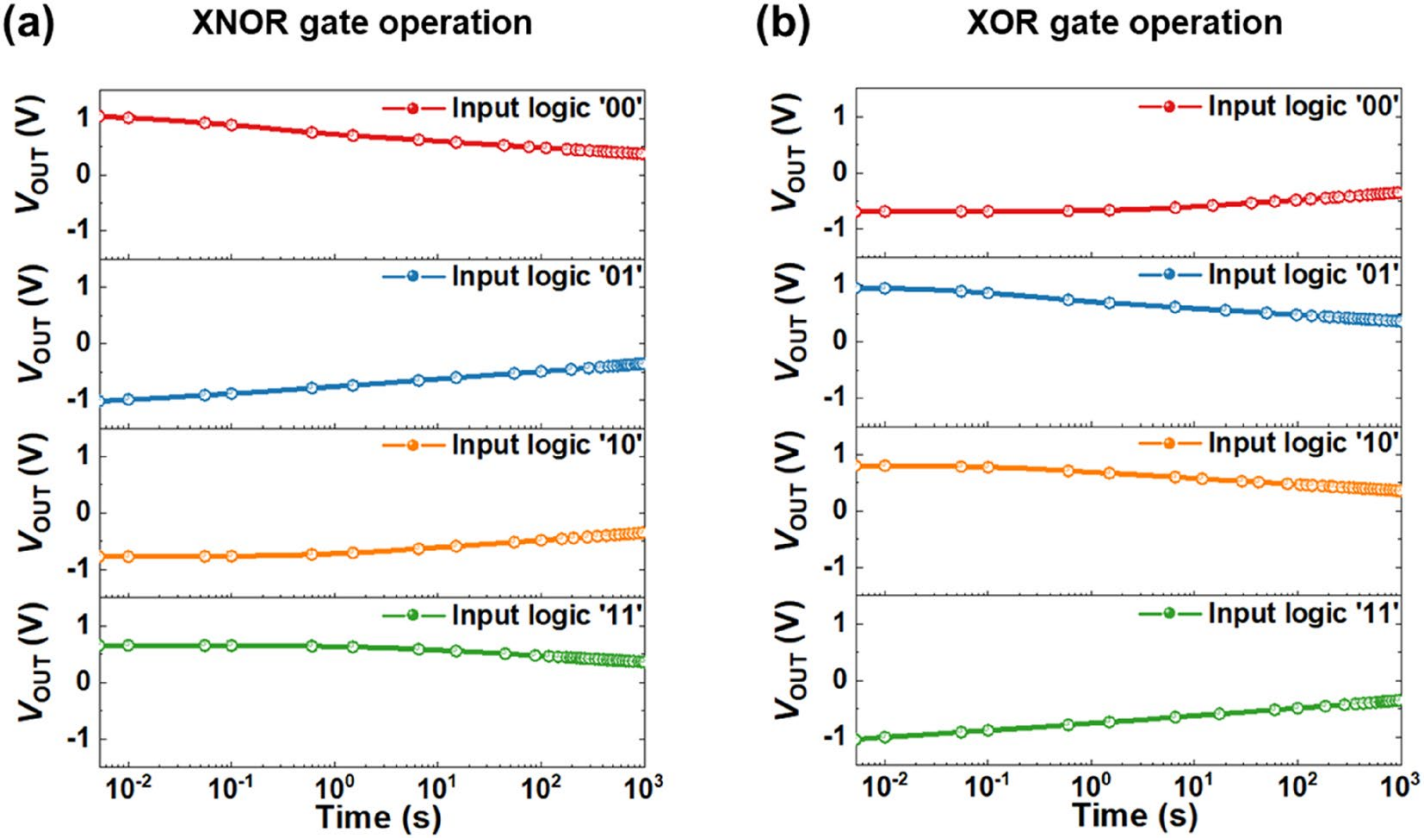
Logic retention characteristics of ULIM cell. *V*_OUT_ versus time during the hold operation for (**a**) XNOR and (**b**) XOR logic gate operations.

We can confirm the change in *V*_OUT_ while increasing the measurement time of the hold operation to 1000 s, to determine the extent to which the output logic of the ULIM cell is retained. Each output logic value of the XNOR and XOR logic gate operations is calculated by applying input logic values with the same voltage conditions as the previous logic operations for 1 ms. After calculating the output logic values, all applied voltages including *V*_SUP_, *V*_PG_, *V*_A_, and *V*_B_, become zero. As shown in Fig. 5, the ULIM cell maintains half of the initial output logic value even at approximately 1000 s for both the XNOR and XOR logic gate operations, revealing an exceptional logic retention capability. The data for other the logic gates (except for XNOR and XOR) provided in Supplementary Fig. 4 also exhibits similar tendencies. Among the silicon-based LIM studies that are fully compatible with existing CMOS processes, no studies with such quasi-nonvolatile characteristics have been proposed thus far. Also, unlike the conventional memory devices that exhibit their memory state when reading voltage is applied, the ULIM cell represents the calculated logic data directly to the voltage value of *V*_OUT_ and retains it for a certain period before the new input is applied, and therefore does not require the sensing voltages for reading. Thus, our ULIM cells suggest the possibility of implementing a new LIM architecture for enabling energy-efficient computing while using existing CMOS processes.

## Conclusions

Through technology computer-aided design simulations, we demonstrated that the proposed ULIM cell can perform all the basic Boolean logic calculations, as well as memory behavior. The triple-gate FBRAMs constituting the ULIM cell can reconfigure the program mode according to the *V*_PG_, and this reconfigurable characteristic at the device level enables the ULIM cell to perform any logic operation from a complete set of two-input or one-input Boolean logic operations. By showing eight different basic Boolean logic operations according to various combinations of *V*_PG_ and *V*_IN_, we proved that our ULIM cell functions as a universal logic cell with a high logic functionality per cell area. Furthermore, the ULIM cells have memory characteristics that can maintain the output logic values based on the excellent carrier storage capability of the triple-gate FBRAMs. These memory characteristics have been demonstrated under a zero-supply voltage, suggesting that the ULIM cells have great potential as energy-efficient hardware.

## Supplementary Information

Supplementary Information.

## Data Availability

All data generated or analyzed during this study are included in this published article and its supplementary information files. The datasets used and/or analyzed during the current study are available from the corresponding author on reasonable request.

## Abbreviations

LIM: Logic-in-memory
ULIM: Universal logic-in-memory
CMOS: Complementary metal–oxide–semiconductor
FBRAM: Feedback random access memories
PG: Program gate
CG: Control gate
*V*_IN_: Input voltage
*L*_G_: Gate length
*L*_GAP_: Separation between the gates
*L*_D_: Drain length
*L*_S_: Source length
*T*_Si_: Channel thickness
*T*_OX_: Oxide thickness
*V*_PG_: Programming voltage
*V*_CG_: Control gate voltage
*I*_DS_: Drain current
*V*_DS_: Drain-to-source voltage
*V*_DD_: Common drain voltage
*V*_SS_: Common source voltage
*V*_OUT_: Output voltage
*V*_A_: Input voltage A
*V*_B_: Input voltage B
*V*_SUP_: Supply voltages
