# Supplementary material for: Universal logic-in-memory cell enabling all basic Boolean algebra logic
Source: Sci Rep. 2022 Nov 22;12:20082. doi: 10.1038/s41598-022-24582-y (PMC9684131; doi:10.1038/s41598-022-24582-y)
Supplement: Supplementary file 1 — Supplementary Information. [file 41598_2022_24582_MOESM1_ESM.docx]

Supplementary information

**Universal logic-in-memory cell enabling all basic Boolean algebra logic**

Eunwoo Baek^1^, Kyoungah Cho^2^ and Sangsig Kim^1,2*^

*^1^Department of Semiconductor Systems Engineering, Korea University, Republic of Korea ^2^Department of Electrical Engineering, Korea University, 145 Anam-ro, Seongbuk-gu, Seoul 02841, Republic of Korea*

**Supplementary Fig 1: Basic symbols of the triple-gate RAM and the ULIM cell**


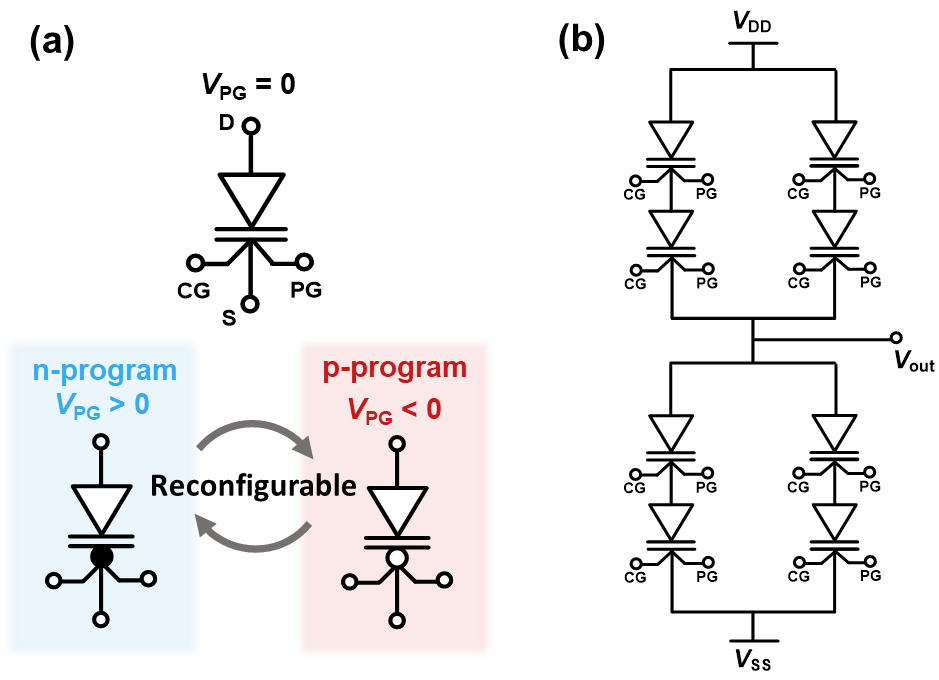


**Supplementary Figure 1.** | (a) The basic circuit symbol of the triple-gate RAMs when *V*_PG_ = 0.0 V. The circuit symbols of n-/p-program modes are expressed below of the basic circuit symbol. (b) Basic circuit of the ULIM cell

**Supplementary Fig 2: ULIM cell demonstrating various logic gate operations**


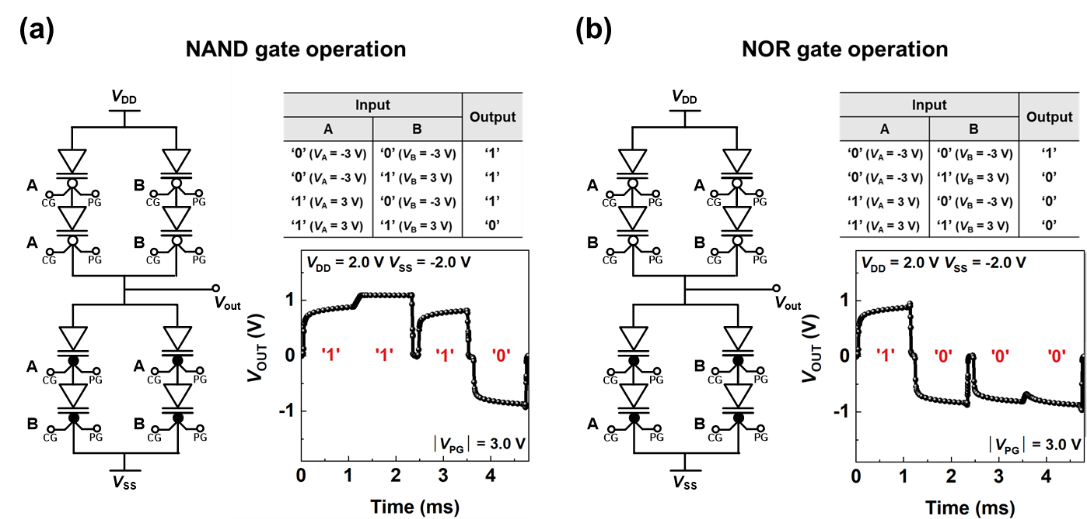


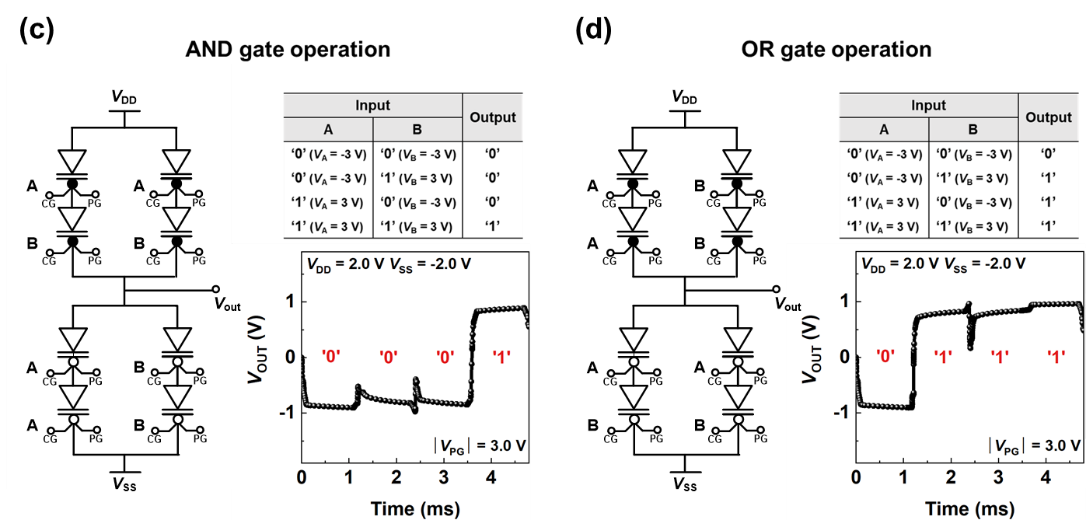


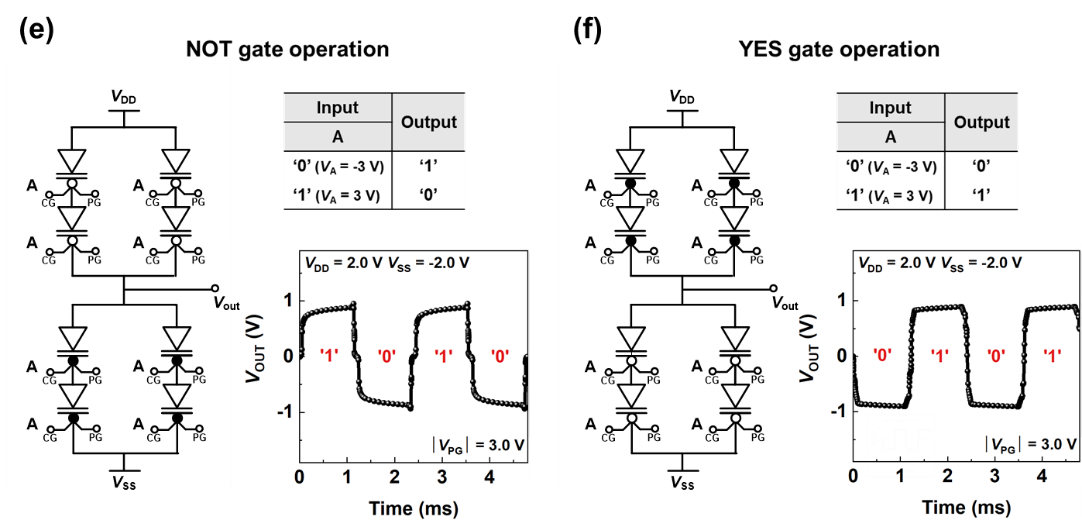


**Supplementary Figure 2. |** Circuit diagrams, truth table and timing diagrams for (a) NAND, (b) NOR, (c) AND, (d) OR, (e) NOT and (f) YES logic gate operations.

**Supplementary Fig 3: LIM operation of the ULIM cell demonstrating various logic operations**


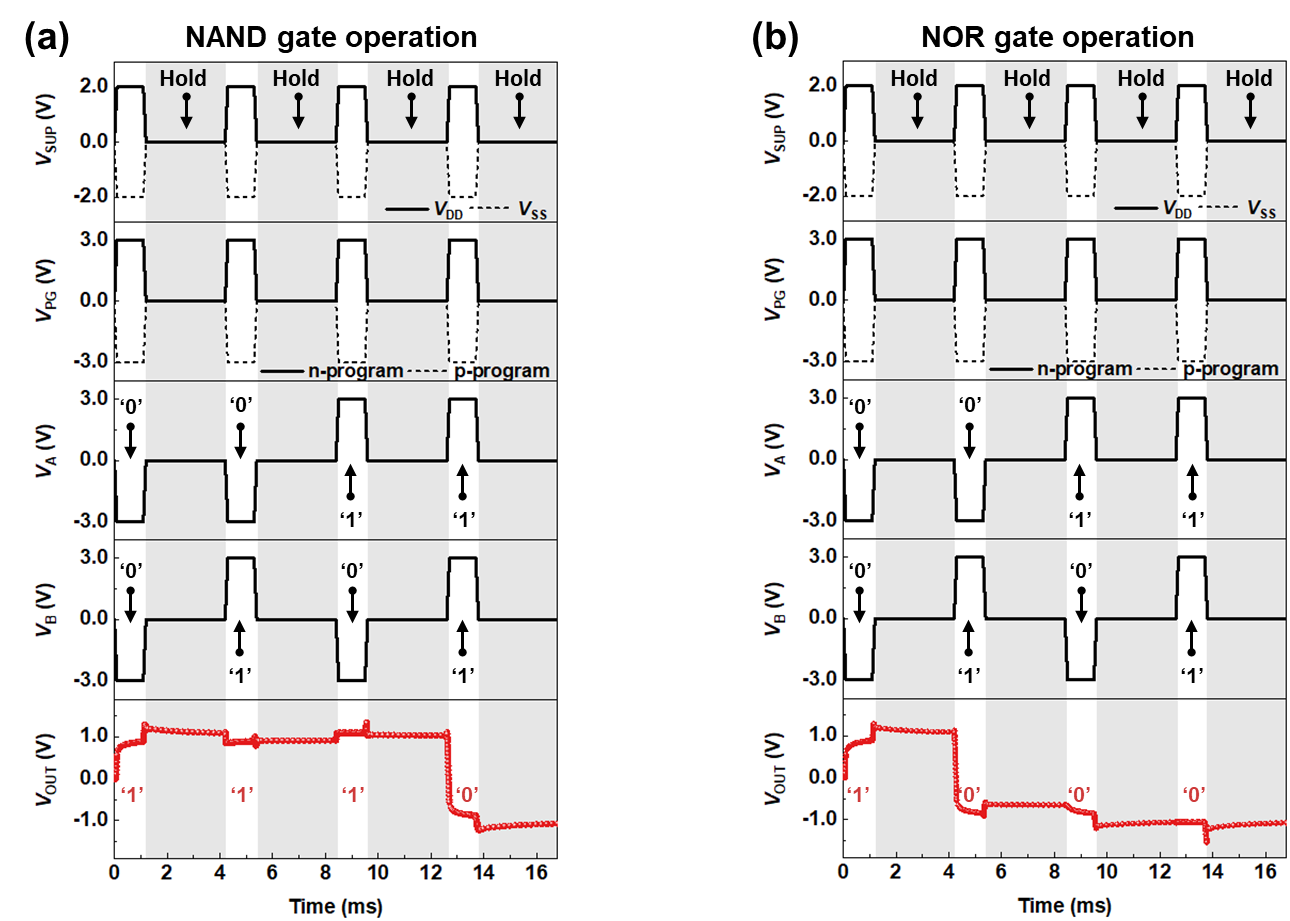


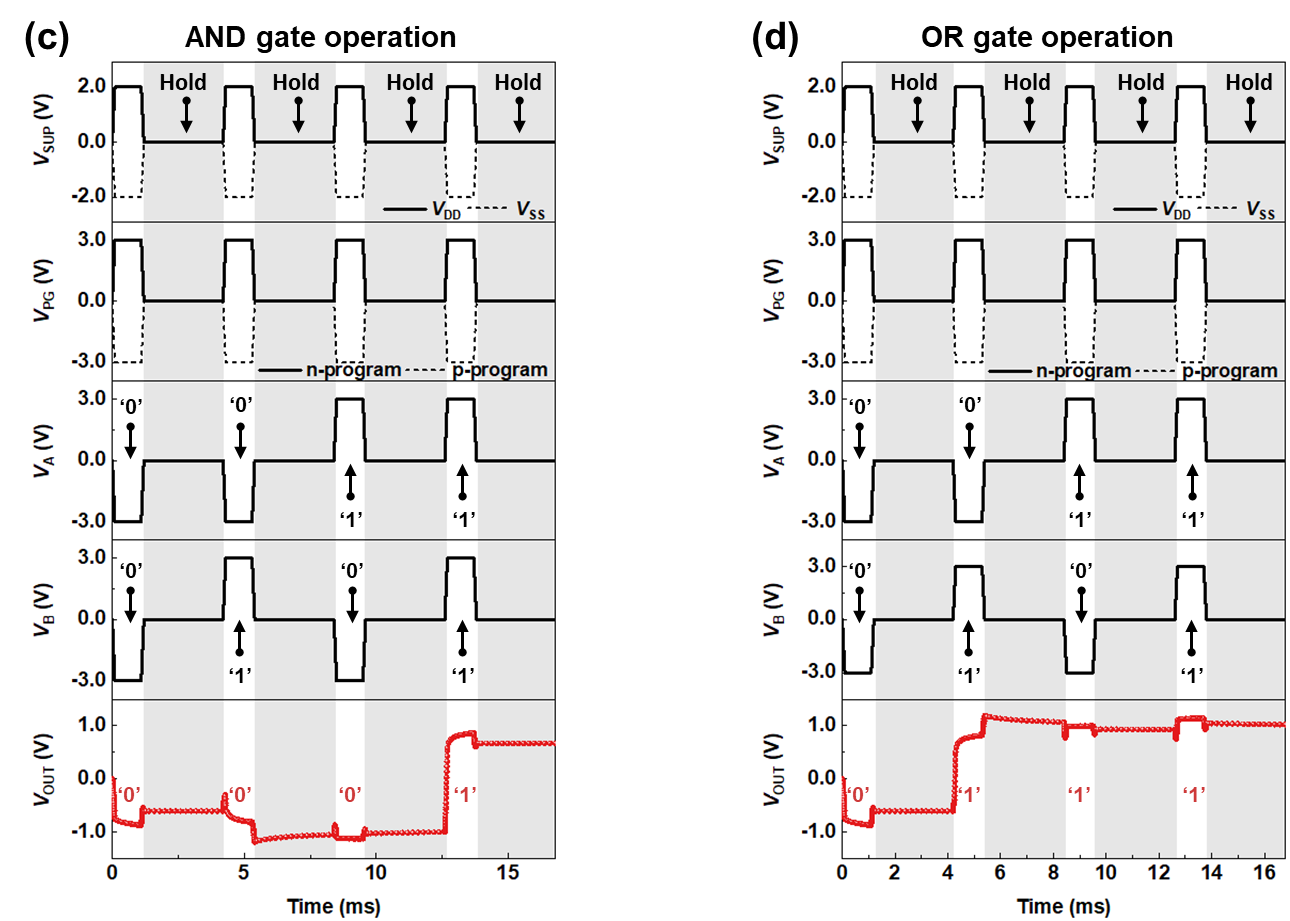


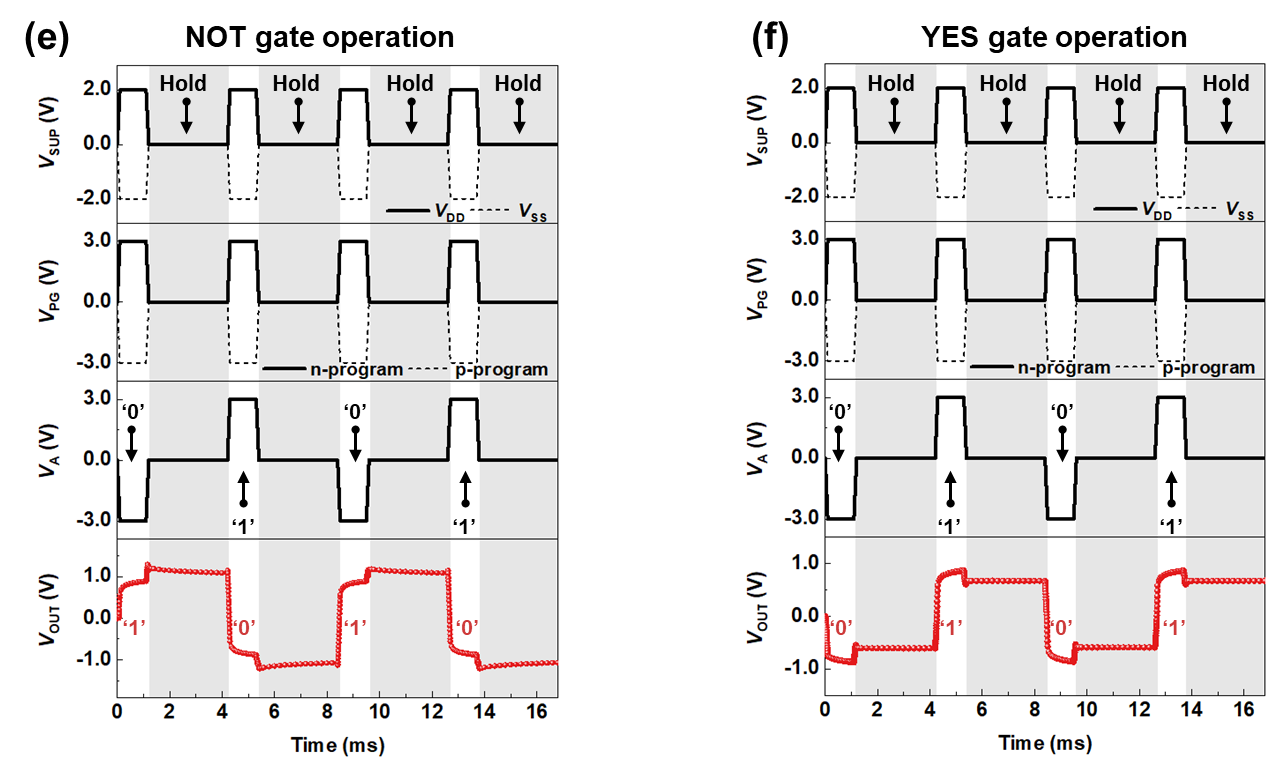


**Supplementary Figure 3.|** Timing diagrams when applying the repeated pulses of the logic and hold for (a) NAND, (b) NOR, (c) AND, (d) OR, (e) NOT and (f) YES logic gates.

**Supplementary Fig 3: Logic retention characteristics of the ULIM cell**


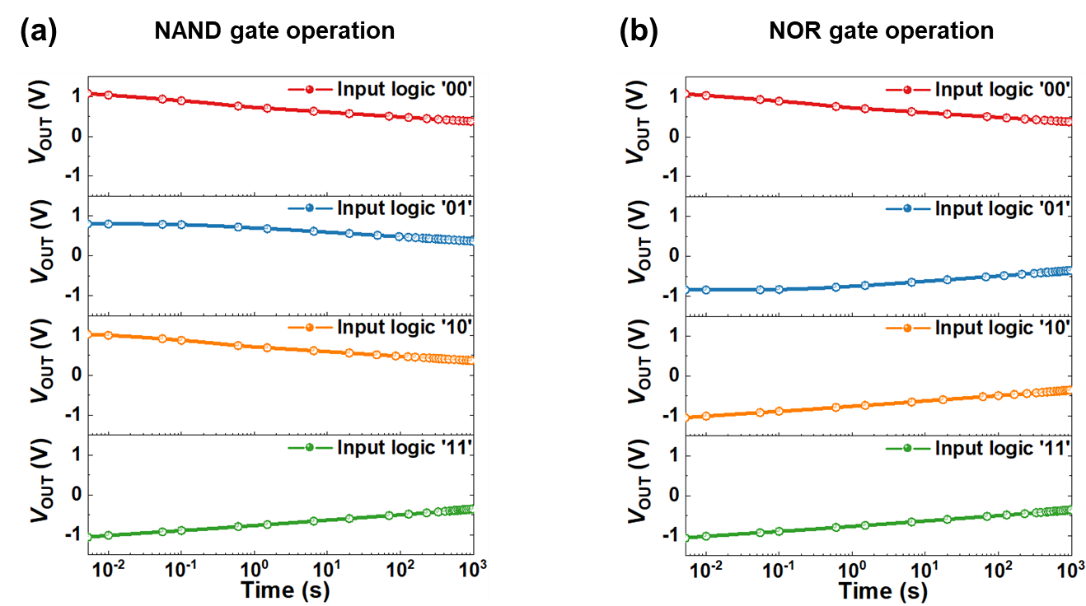


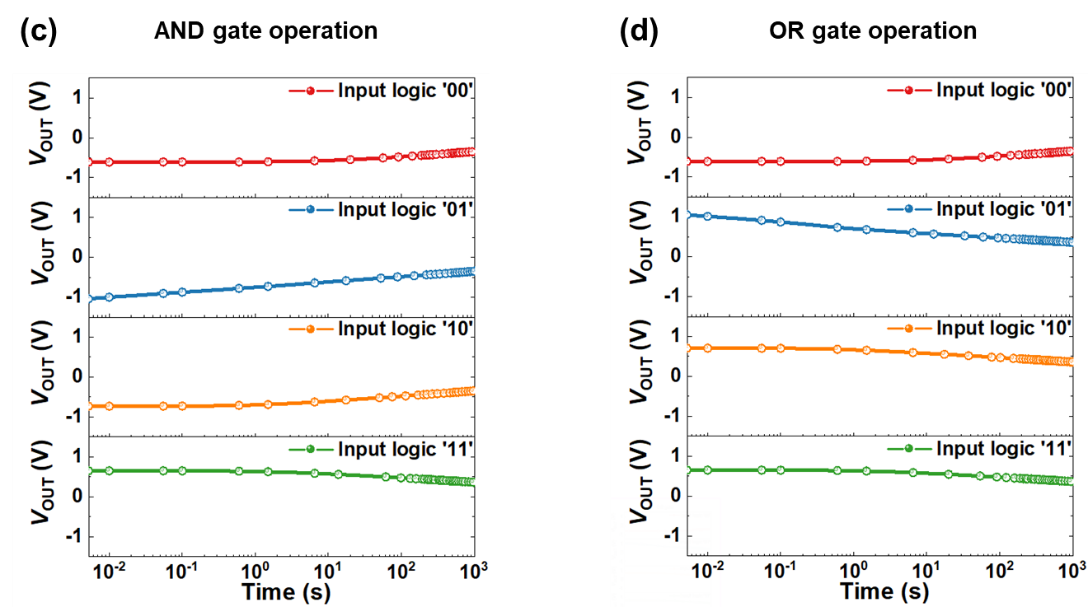


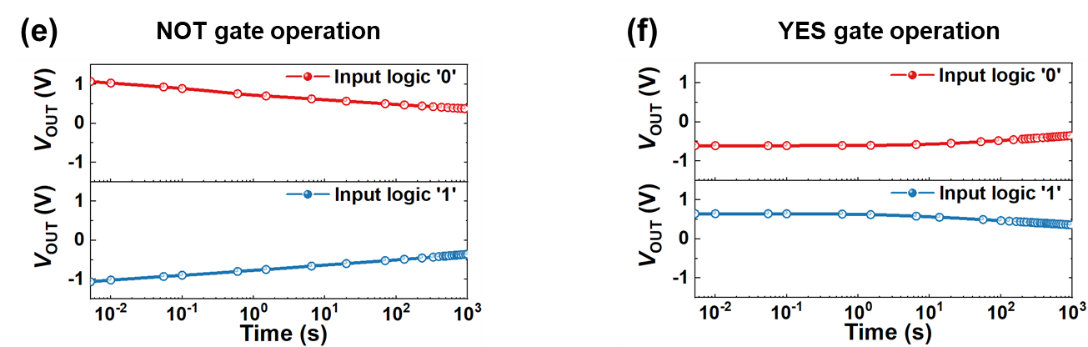


**Supplementary Figure 4.|** *V*_OUT_ versus time during the hold operation for (a) NAND, (b) NOR, (c) AND, (d) OR, (e) NOT, and (f) YES logic gate operations.
